# Comparison of the noise produced by the polymer and bronze P1566 propellers in the cavitation tunnel

**DOI:** 10.1038/s41598-023-47342-y

**Published:** 2023-11-15

**Authors:** Piotr Szymak, Andrzej Grządziela, Krzysztof Naus

**Affiliations:** 1 Department of Naval Automatics, Polish Naval Academy, Śmidowicza 69, 81-127 Gdynia, Poland; 2 Institute of Ship Building, Polish Naval Academy, Śmidowicza 69, 81-127 Gdynia, Poland; 3 Department of Navigation and Hydrography, Polish Naval Academy, Śmidowicza 69, 81-127 Gdynia, Poland

**Keywords:** Civil engineering, Electrical and electronic engineering, Mechanical engineering

## Abstract

The paper presents the selected results of the international project called Nextprop, carried out in the framework of the European Defence Agency, which main goal was to examine elastic polymer propellers. The article includes the results of the comparative analysis in terms of the noise produced by two propellers: (1) the classical one made from Nickel Aluminium Bronze (NAB) and (2) the novel elastic counterpart manufactured from polyamide. The measurements were taken in the CTO cavitation tunnel in Gdańsk, Poland. The obtained results allow the following highlights to be formulated: the novel flexible propellers seem to be a promising solution for the new ships, especially in terms of energetic efficiency and produced noise, i.e. lower sound pressure level has been obtained for the new polymer propeller and larger advance speed for the same rotational speeds has been received for the new propeller compared to the classical metal counterpart. At the beginning, the introduction with the results of state-of-the-art analysis is presented. Then, the description of the measurement stand and examined propellers with seizing conditions is included. Next, the obtained results are illustrated and discussed. At the end, the conclusions with the draft of the future research are presented.

## Introduction

Propeller noise is usually a factor in the acoustic signature of ships, which is why navies are interested in this research area. Knowledge of acoustic signatures allows for quick recognition of ship classes and the use of artificial intelligence in destroying the precisely recognized surface and underwater objects by sea mines. In the civil maritime industry, the interest in the noise emitted by propellers results from the need to reduce the harmful effects of cavitation, extend the lifetime, and increase the comfort of sea travel. Despite advanced numerical tools that allow predicting the acoustic signatures of propellers operating at the ship’s hull in the design phase, model tests are still the most reliable and commonly used research method. The test results could be better, but they require experience to interpret the data at the scale of the model and obtain consistent predictions of what is a real challenge.

Marine propellers have many design and material solutions for technical needs and operational scenarios. Background information on the applications, construction, geometry, and performance of ship propellers are described, among others, in^1^ and^2^. Marine technology mainly focuses on the durability of solutions, energy efficiency, and, recently, more often, on reducing emitted noise. In the case of military applications, the main goal is to meet tactical requirements and operational scenarios. Depending on the class of ships, Naval propellers can have from 3 to 9 wings and operate in non-cavitating, cavitating, and even supercavitating rotational speed ranges. Due to the need for a low Sound Pressure Level (SPL) requirement, propellers for submarines are made with the number of wings usually 7. Currently, new propulsor solutions like pump-jet type propulsors or similar^3^ are being developed, but all of them should meet the following requirements^4^:

- Low acoustic signatures;
- Wide operational window covering endurance, speed, and depth;
- High manoeuvrability;
- Best possible form to achieve a low hydrodynamic resistance and a smooth hull wake;
- Propeller with low excitation level to noise;
- Propeller free of cavitation for operational speeds;
- High-efficiency propeller.

In the case of using propulsors for underwater drones, one of the possible solutions is 4-blade propellers. Due to operational scenarios, the operating range is usually expected to be below the critical cavitation number to obtain a quiet and effective drive. Using composite or polymer materials allows these features to be achieved in a highly satisfactory manner. Therefore, there is a need to assess the impact of the materials used on the acoustic emission of the operating propeller and the transmission of vibroacoustic energy transferred through the shaft line to the marine environment. This first aspect is the subject of this work.

The regulations on propeller noise measurement are successively defined every few years by the International Towing Tank Conference (ITTC). Recommended procedures and guidelines are included in the ITTC Quality System Manual. The last recommendations on model scale propeller noise measurement were updated and proposed in 2021^5^. The noise is mainly used to predict the natural noise emission for the full-scale propeller. The procedures focus on measuring the propeller’s cavitation noise. Still, it is also applicable for measuring other forms of acoustic emission, including determining the noise of non-cavitating propellers. The guidelines present the test set-up, including requirements for the propeller model, wake generation, and sensitivity of applicated hydrophones.

Moreover, ITTC gives recommendations on background noise measurement, data acquisition and processing, and seizing the acoustic transfer function of the facility. The recommended procedures and guidelines were also applied to the measurements carried out in the Nextprop project.

Moreover, precise requirements exist for test conditions, instrumentation, procedures and scaling methods. An essential advantage of this document is the chapter UNCERTAINTY and VALIDATION, which indicates the primary sources of measurement errors and the way of performing and presenting the uncertainty assessment methodology. It is a helpful guide that allows you to achieve repeatable measurements without committing methodological errors. Noise measurement recommendations are also included in documents from other organizations, such as ISO and IMO^6–8^.

Scaled experimental and numerical simulations are used in ship propellers’ hydrodynamic and hydroacoustic designs. Despite the use of advanced numerical tools for modelling fluid dynamics, experimental methods implemented first in cavitation tunnels, then in towing tanks and finally during sea trials allow us to obtain more accurate results. The recommendations of the ITTC committee are usually guidelines for testing both cavitating and non-cavitating propellers^9,10^. Experimental research requires many tests and the acceptance of distortions and simplifications.

The literature review was performed for the following areas of interest:

- Preparation of the measurement methodology using hydrophones.
- Measurement in the cavitation conditions as a specific case of the noise measurement.
- Analysis of proposed solutions of non-metal propellers.

First, an essential step in measurement is the preparation phase. Some guidelines can be found in the literature. In 1987, Matsuda, Boucheron and Ohta researched the effect of cavitation channel walls on propeller noise^11^. Test results indicated a difference of 15 dB in SPL between comparing the experimental results with the panel method due to the reflection of the acoustic waves from the wall^12,13^. In 2019, Sharma, Skvortsov, MacGillivray, and Kessissoglou examined sound absorption by rubber coatings with periodic voids and hard inclusions^14^. They analyzed the generated sound pressure level changes for non-cavitating and cavitating marine propellers. It was indicated that the increase in sound pressure at the transition to the cavitation state ranged from 10 dB to 30 dB. The assessment of the emitted noise, as the overriding criterion, was tested on the impact of the increase in rotation in the non-cavitation zone at a constant inflow (1.5 m/s) and a constant pressure of 90 kPa^15^. They pointed to an increase in SPL in 1/3 octave bands from 3 to 7 dB.

The analysis of the facility’s cavitation tunnel’s impact as an acoustic environment for estimating the propeller source levels in ideal free field conditions is presented in the paper^16^. The authors indicate that this is done by measuring the object’s transfer functions and applying such functions as corrections to the measured noise spectra. The paper addresses this problem by discussing the techniques, advantages and possible issues in detail. It was shown by example that transmittances measured with an electronic noise emitter often have unrealistic characteristics (e.g. anomalous peaks and troughs). A 1/3 octave band representation was proposed to reduce effects irrelevant to the study of the system’s response to random excitation, such as cavitation noise. It was indicated that tests with multiple source positions and/or a moving emitter should be used to measure the transfer function in a cavitation tunnel, disregarding phenomena such as wave interference. From this point of view, increasing the number of items tested improves the results.

The work^17^ presented model-scale tests carried out in a cavitation tunnel on a model propeller of a research catamaran. Testing was conducted using a 2D wake-up screen to approximate the actual state of the vessel. The paper details the experimental setup and results and compares them with full-scale measurements regarding cavitation and underwater noise emissions. Emission noise measurements were carried out with three hydrophones and a Bruel & Kjaer type 2635 charging amplifier. The track simulation in the tunnel was achieved using 2D wire mesh wake-up screens due to the limited dimensions of the cavitation tunnel. Similar results are presented in the work published by Atlar et al. in 2001^18^. The paper presents the results of cavitation tunnel tests carried out on the model of the 4-blade and silenced CPP propeller of the Fishing Research Vessel and noise measurements with full-scale validation tests of this propeller. The study also includes measures of model propeller noise levels and their analysis. Accurate prediction of full-scale propeller noise based on model tests carried out in a cavitation tunnel is only possible with detailed knowledge of the effect of proximity to tunnel walls and other factors. Determining the correlation coefficients to be applied to model measurements requires an extensive program of model and full-scale testing, which is risky anyway. Depending on the tunnel type, propeller noise will require special attention due to its reverberant nature. Due to the research carried out on a model scale, the preparation of the measurement methodology should be exact and consistent with international standards.

Very often, hydrophone measurement is devoted to cavitation phenomena, which can be treated as a specific case of seizing noise produced by propellers. Hallander and Grekula presented interesting research results in the report published in 2017^19^. The tests included acoustic emission measurements in a typical model hull setup with model two propellers in a cavitation tunnel. The research was focused on identifying the threshold of the acoustic level at which cavitation erosion occurs. The results indicated a threshold ambiguity depending on the geometric features of the propeller.

Widjiati et al.^20^ presented a method for obtaining reliable measurement results, including calibration measurement and minimizing system and environmental acoustic noise in a cavitation tunnel for propellers operating without and with cavitation. Analyses of the characteristics of the measurement results in the time-frequency domain were performed to detect when and what type of cavitation noise occurs in any conditions. The results in acoustic data signals were obtained in various environmental conditions by changing the water pressure, flow speed and propeller rotational speed.

Felli M., in the Project’s HTA Report^21^, indicated that the latest generation of extensive cavitation testing equipment enables these tests to be performed in a realistic three-dimensional wakefield of complete ship models. It has been noted that model-scale noise measurements are typically performed at facilities with significant background noise and reverberant test sections. Separating each “false” contribution from the overall noise level is difficult. Instead, it is possible to correct the contribution of unwanted sources by adopting noise reduction techniques. The paper explains the hydrodynamic aspects of the impact on the noise structure, i.e. noise generated by tributary disturbances, wake evolution mechanisms and propeller-rudder interaction. Most measurement procedures for noise measurement refer to the cavitation phenomenon, but these procedures also work well in testing non-cavitating propellers. Guidelines specifying how to measure noise emitted by propellers and validating the results can be found in these publications and adopted to the cases without cavitation.

The interesting approach to measuring the underwater noise generated by a ship is included in^22^. The Authors attempted to quantify the source strength using onboard pressure sensors near the propeller. In the study, a beamforming method was used to estimate the source strength of a cavitating propeller. The method was validated against a model-scale measurement in a cavitation tunnel, which showed good agreement between the measured and estimated source levels. The method was also applied to a full-scale measurement, in which the source level was measured using an external hydrophone array^22^.

Considering the proposed solutions for non-metal propellers, several publications can be found. Regarding the new constructions of marine propellers, recently, it can be seen an increasing number of research on non-metal propellers, especially composite ones^23–26^. The research is focused on two main areas:

- Comparison tests on composite propellers vs. classical ones fabricated from different types of steal,
- Designing, optimization and manufacturing processes of the new composite propellers.

The first group of the research aims to evaluate the advantages and disadvantages of the new composite propulsion compared to their classical counterparts. While the goal of the second area of the study is to share knowledge about the latest technology of composite propeller manufacture with partial goals like designing rules, optimization, etc.

There are several examples of comparison tests on composite propellers vs classical ones fabricated from different types of metal. In^25^, the examination of tip deflection and cavitation symptoms based on the measurement of propeller models in the test tank and FSI analysis has been presented. While in^26^, the evaluation of vibration and cavitation erosion based on measurement is described in detail. Exciting results of an examination of efficiency and vibration induced by classical and composite propellers driving large ships in open water are included in the report of the ship manufacturer^24^. Based on the presented research, two main advantages can be formulated for the new composite propellers:

- Higher energetic efficiency, resulting in a lower cost of exploitation and longer cruise,
- Lower noise generated by the propeller and/or transferred from the engine room via a shaft to the outside underwater environment.

Noise caused by operating machines inside the ship affects the working comfort and social conditions of ship crews. Although ship propellers are the least harmful factor, it should be noted that the precision standards of noise limits were developed in 1981^27^. The recommendations contained in this document precisely define the tolerated values for both social and technical rooms. The threshold values indicated noise acceptance levels for ship spaces in Octave Band Center Frequency.

As can be seen, the new flexible propellers offer quite interesting capabilities, which should be further tested and improved. Therefore, the paper undertakes the problem of comparative analysis, especially in terms of the noise produced by two propellers: (1) the classical one made from Nickel Aluminium Bronze (NAB), and (2) the novel elastic counterpart manufactured from the polyamide.

Concerning the requirements of the Navies, the standards regarding the permissible or recommended acoustic emission by the propeller are not public documents, and each Navy and even shipyard has its own requirements and test standards both for propellers operating in cavitation tunnels and during sea trials for the entire propulsion system. The main goal is to obtain the lowest possible SPL generated by the propulsor within the selected rotational speed or speed range. It should be noted that the publications on this subject^28–30^ refer to the SPL generated by a moving ship rather than to the precise identification of the noise source, which is the propeller.

The novelty of the research presented in the paper involves examining the impact of the material used for the propeller’s blade manufacturing on the acoustic emissions of the working propeller. In this part of the work, all acoustic disturbances originating from the drive were removed, and the focus was only on the impact of the flexible material on the noise emitted by the operating propulsor in the rotational speed range below the cavitation critical number.

In the next section, the measurement stand and examined objects are presented. Then, the results of the tests and measurements performed are described and discussed. In the end, the conclusions from the comparative measurements are inserted.

## Material and methods

### The measurement stand

As mentioned earlier, the paper includes the results of the hydroacoustic measurements of the propellers using the cavitation tunnel installed in the CTO. The total dimensions of the tunnel are almost 24 m wide and 12 m height. The measurement chamber has a length of 3080 mm and a cross-section 800 mm x 800 mm. It enables the flow of water with a maximum speed 20 m/s. The tunnel equipment includes, among others, a system based on high-speed cameras enabling the observation and recording of cavitation, dynamometers, a system for measuring pressure pulses on the hull surface, as well as a laser anemometer (LDA) for measuring the velocity field, e.g. in the propeller circle.

**Figure 1:**
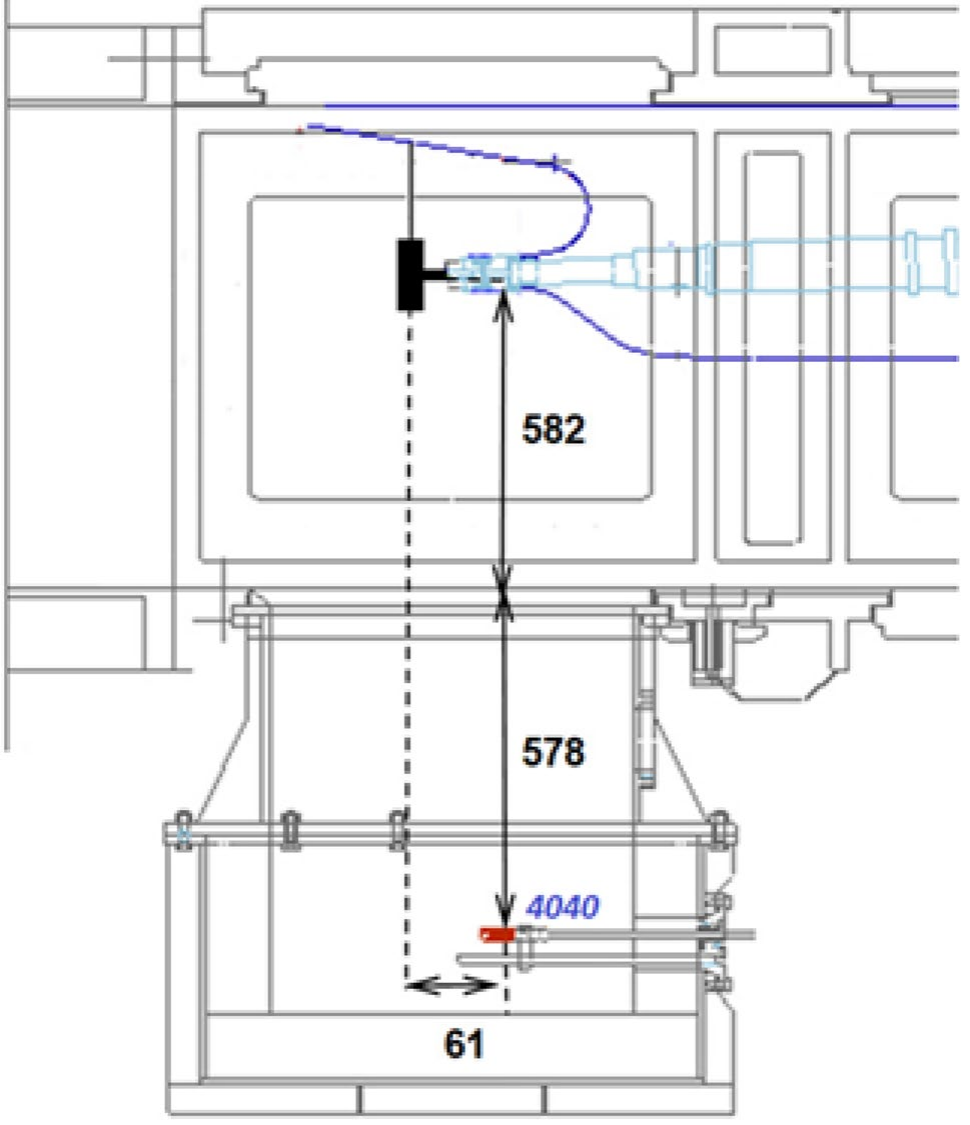
Localisation of the propeller and hydrophone in the measurement chamber of the cavitation tunnel in the CTO.

**Figure 2:**
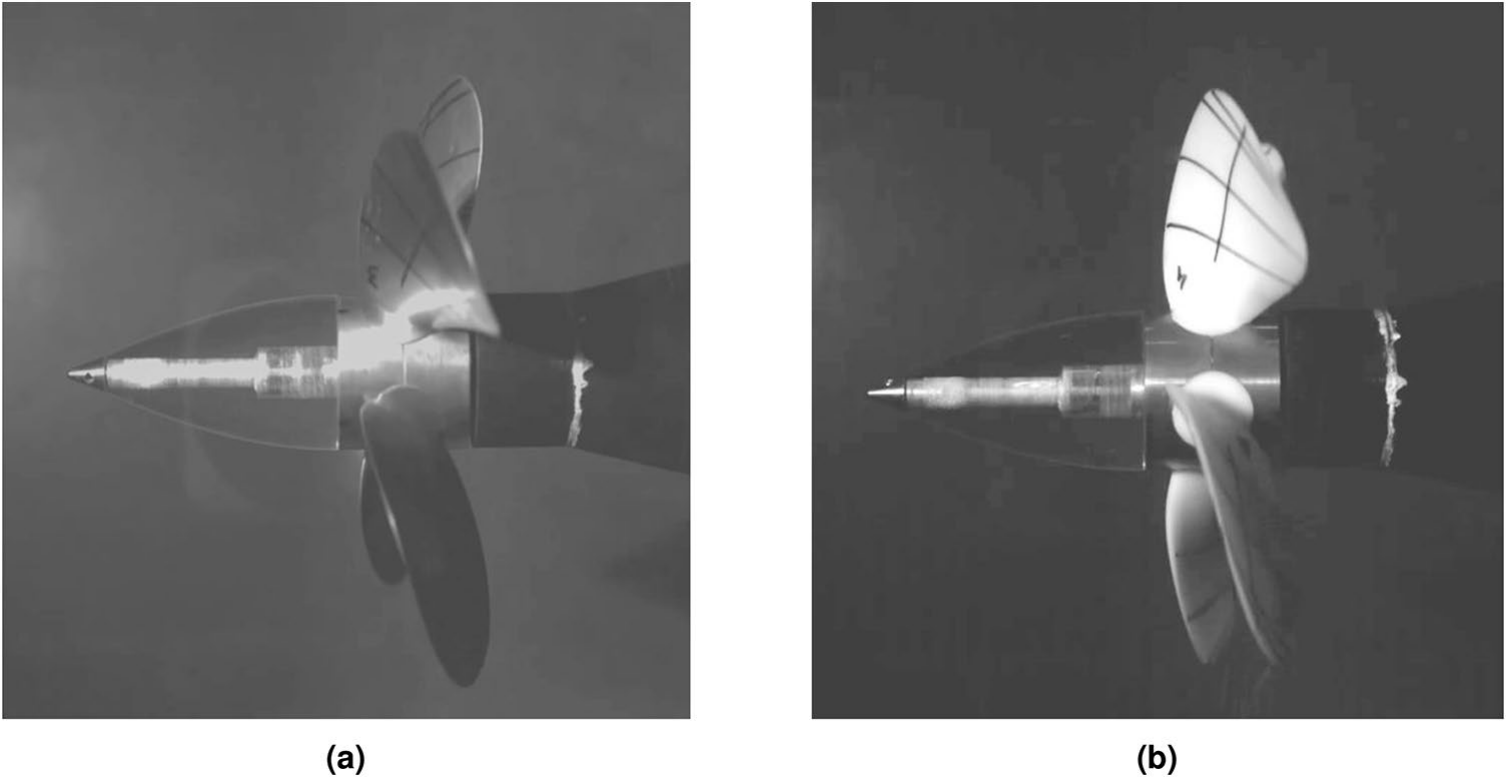
The NAB (**a**) and polyamide (**b**) propellers during their tests in the cavitation tunnel in the CTO.

The experiments were done in the measurement chamber (Fig. 1). The ship model with the propeller mounted on the aft is placed in the upper part of the chamber. While the hydrophone, registering noise generated by the propeller and other equipment, is installed in the lower part of the chamber, i.e. in the special additional chamber whose walls are lined with an absorbing material that suppresses possible reflections from the walls. In this experiment, the three following cases were considered:

1. Only the sound produced by the rotating shaft without any propeller was recorded.
2. The NAB propeller was mounted and produced noise was registered (Fig. 2a).
3. The sound generated by the polyamide propeller was recorded (Fig. 2b).

It is worth mentioning that in all the listed cases, also additional noise is emitted by other equipment, especially pumps responsible for water flow in the cavitation tunnel.

During all the analyzed cases, a 4040 type hydrophone from Teledyne company was used for the produced sound registration. As mentioned earlier, the hydrophone was mounted in the additional chamber covered by absorbing material to avoid possible interferences and reflections. Based on the previous measurements, such an approach ensures minimizing possible disturbances. However, it was challenging to prevent noise generated by additional equipment in the cavitation tunnel, mainly destined for the water flow. Unfortunately, such equipment has to work continuously.

All the measurement equipment was supplied from the independent battery power supply system to avoid any disturbances from the power network.

### The examined propellers

The examined propellers were manufactured from different materials but using the same four-blade propeller geometry identified with code P1566. It is worth mentioning that both propellers are composed of a hub and four blades mounted to the hub. In both cases, the hub was made from bronze type BA1032. The blades in the rigid propeller were made from bronze type BA1032, while the blades in the elastic propeller were produced using Technyl A205F natural polyamide-PA66. Their main features are summarized in Table 1.

**Table 1:** Main features of the materials used to produce rigid and elastic propellers.

	BA1032	PA66 (in water)
Elasticity modulus *E* [Gpa]	95.772	1.88
Tensile strength *R*_*m*_ [Mpa]	756	82
Elongation *A* [%]	23.5	89
Density [kg/m^3^]	7650	1140
Water absorption [%]	0	3,25

The preferred method for rigid propeller manufacturing was CNC machining and moulding for blades of the elastic propeller. A 5-axis CNC machine with a tool travel range of 1016 x 508 x 762 mm for longitudinal, transversal, and vertical direction, respectively, was used to produce the propeller blades and the hub.

### The seizing conditions

**Figure 3:**
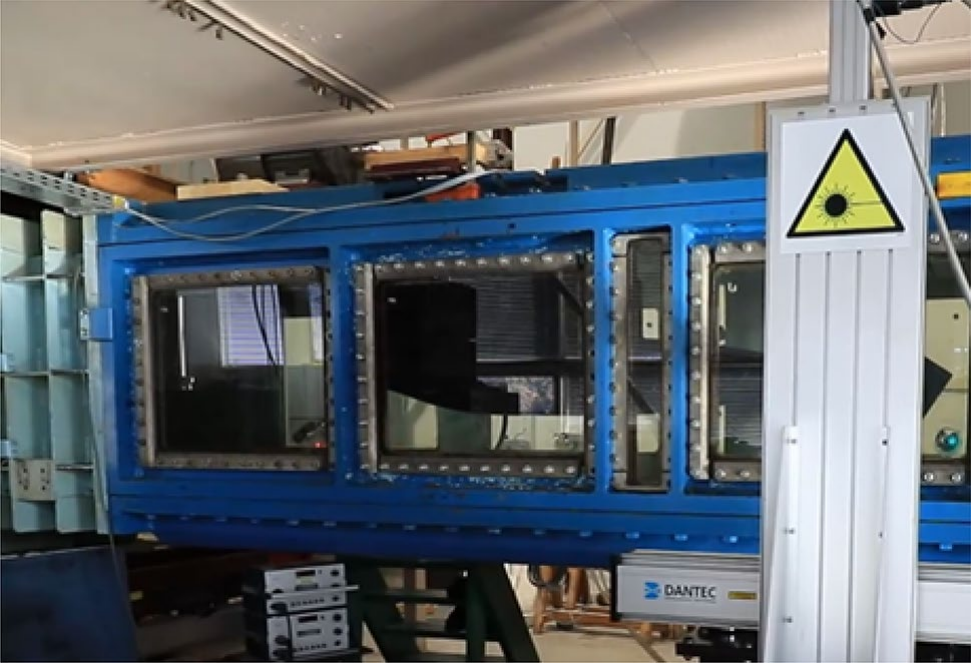
The view of the chamber of cavitation tunnel in CTO.

**Figure 4:**
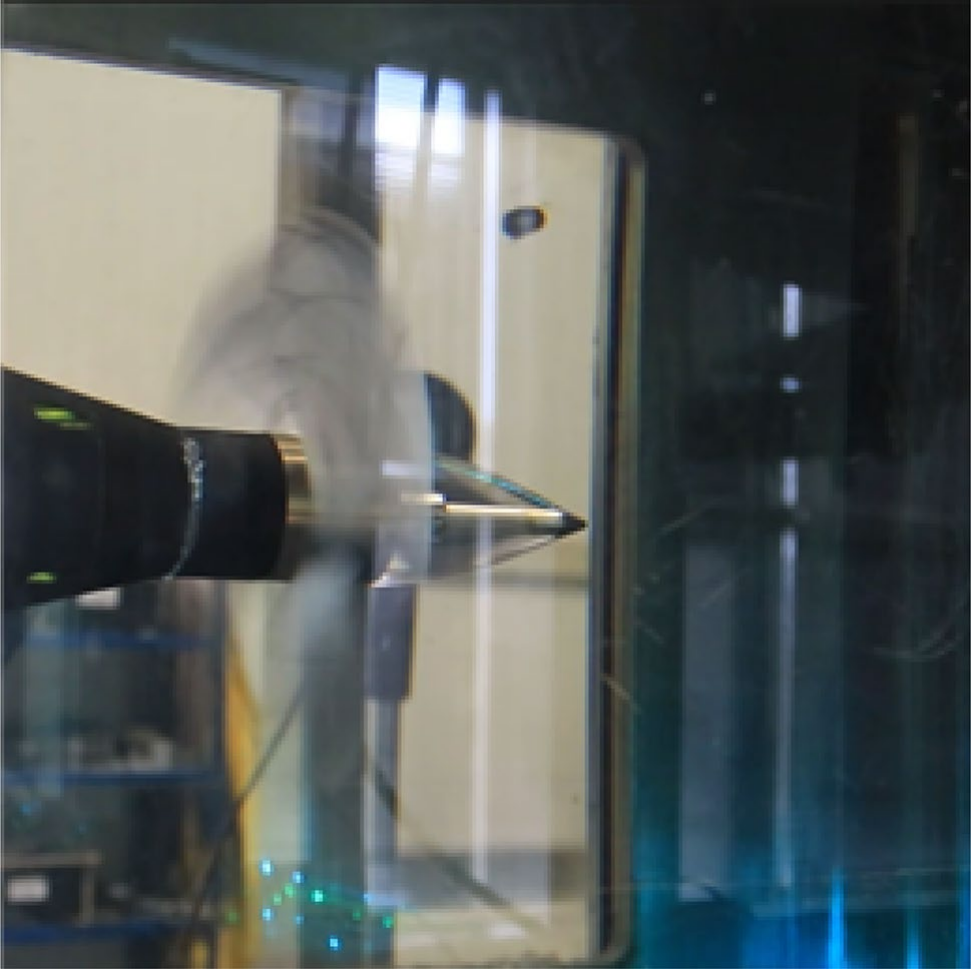
The view of the rotating propeller in the cavitation tunnel in CTO.

In Fig. 3, the cavitation tunnel installed in CTO is visualized. As mentioned in the previous section, three cases of different shaft loading with (Fig. 4) and without rotating propellers were examined. Moreover, the shaft and propellers were tested for the following rotational speeds: (1) 7 r/s, (2) 9 r/s, (3) 11 r/s, and 20 r/s (except polyamide propeller due to the risk of its damage). For each rotational speed, the thrust was adjusted to achieve the required thrust coefficient values: 0.5, 0.4, and 0.2. For the highest rotational speed, the thrust coefficient values of 0.5 and 0.4 were omitted due to high load, exceeding the dynamometer range, and possibly also the blades’ material capacity.

**Figure 5:**
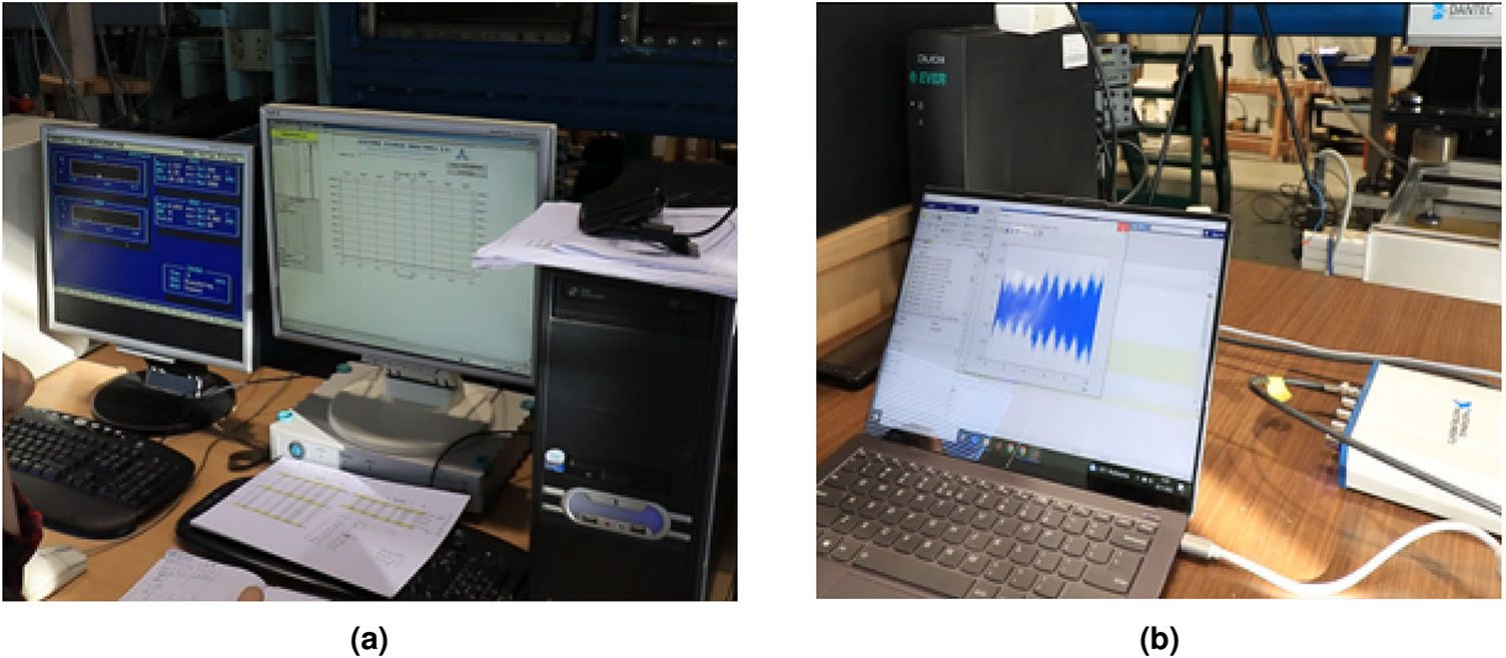
The view of the stands for (**a**) thrust and rotational speed measurement, (**b**) hydroacoustic signature seizing in the CTO cavitation tunnel laboratory.

The measurements were conducted in the following way. After mounting a specific propeller or dismounting (tests for only the shaft), the desired one of four rotational speeds was set. Then, the water flow was increased step by step by observing the produced thrust (Fig. 5a). After achieving stabilized rotations of the propeller and flow of water in the cavitation tunnel, the hydroacoustic signature was registered for 10 seconds using National Instruments DAC connected to the PC with Matlab application (Fig. 5b).

The measurements for the specific shaft loading case and rotational speed were finished after receiving zero thrusts. i.e. the thrust produced by the propeller was balanced by the hydrodynamic damping force. The seizing with the only shaft was conducted for the water flow speeds obtained for the NAB propeller.

### Qualitative and quantitative measures

To make a qualitative evaluation of the obtained results of measurement, the following measures have been used:

1. The time course of voltage signal from the hydrophone *S*.
2. The spectrum of the voltage signal from the hydrophone expressed in decibels $S_{dB}$.
3. The spectrum of the peaks of the voltage signal expressed in decibels $S_{dBpeaks}$.

The main parameter describing acoustic waves is a Sound Pressure Level (SPL) which is expressed in the Pascal [Pa]. The SPL is proportional to the voltage signal from the hydrophone *S*, for the flat frequency characteristics of the hydrophone receiving sensitivity, i.e. when the sensitivity is constant for different frequencies. Used in the measurements hydrophone TC4040 has flat characteristics of the receiving sensitivity for the frequency band from 0 to 80 kHz^31^. Therefore, for the band from 0 to 80 kHz, we can assume that SPL is proportional to the voltage signal from the hydrophone *S*.

The first qualitative measure, i.e. the time course of the voltage signal from the hydrophone *S* allows us to observe the dynamics and strength of the acoustic wave in time. The second qualitative measure, i.e. the spectrum of the voltage signal from the hydrophone expressed in decibels $S_{dB}$ enables us to see the level of various harmonic frequencies of the acoustic wave. In the second case, the voltage signal had to be converted into decibels due to large differences between amplitudes of various harmonic frequencies using the following formula:

1 $$\begin{aligned} S_{dB}(i)=20 \log _{10} S(i) \end{aligned}$$

Where:

*i* - the ordinal number of the next harmonic of the signal spectrum

To analyze the most significant frequency components, the spectrum $S_{dB}$ was searched to find harmonic components with the largest amplitude called peaks. Signal peaks are understood as local maxima determined in that case using the *findpeaks* Matlab function with the *MinPeakDistance* option equal to 10. In general, the *findpeaks* function compares each frequency component $S_{dB}(i)$ with two adjacent components, i.e. $S_{dB}(i-1)$ and $S_{dB}(i+1)$, and search the component which is larger from the adjacent ones. The additional option *MinPeakDistance* enables us to eliminate peaks that are situated too close to themselves. If the peaks are located at less or equal distance, considering the following elements of a vector $S_{dB}$, the peaks with smaller amplitude are removed.

To make a quantitative assessment of the obtained results, the average signal levels $S_{avg}$ of the hydroacoustic signal for different frequency bands from $f_1$ to $f_2$ frequencies were calculated using the following formula:

2 $$\begin{aligned} S_{avg}=\frac{\sum _{i=k_1}^{k_2}{A_i}}{k_2-k_1} \end{aligned}$$

Where:

$A_i$ - the amplitude of the *i*-th harmonic component of the signal spectrum

$k_1$ - the ordinal number of the harmonic component placed on the frequency $f_1$

$k_2$ - the ordinal number of the harmonic component placed on the frequency $f_2$

## Results and discussion

In the first subsection, the results of the initial phase of the research are included. As mentioned earlier, in the beginning, the water flow had to be increased step by step with an observation of the produced thrust to achieve the balance between the thrust and hydrodynamic damping force for the desired rotational speed. After achieving stabilized rotations of the propeller and flow of water in the cavitation tunnel, the proper measurements can be conducted.

In general, the measurements were conducted according to the ITTC Recommended Procedures and Guidelines^5^. The recommendations were implemented by qualified and experienced personnel responsible for the maintenance of the cavitation tunnel in the CTO. The constant experimental conditions, especially the water flow in the cavitation tunnel, were ensured by appropriate sensors and actuators. Always, the next step of measurements was carried out after receiving stable conditions of the previous stage, e.g. measurement of the noise was conducted after obtaining balance between the thrust and hydrodynamic damping force for the desired rotational speed and stable water flow. To minimize the influence of other noise sources, a simple procedure was applied: all the electric devices located close to the cavitation tunnel were switched off during hydroacoustic measurement. Unfortunately, many pumps and motors must work to keep the constant experimental conditions, especially water flow in the cavitation tunnel.

In the following three subsections, the results of the proper measurements, consequently for the shaft without propeller and then NAB and polyamide propellers.

### Initial phase of tests

**Figure 6:**
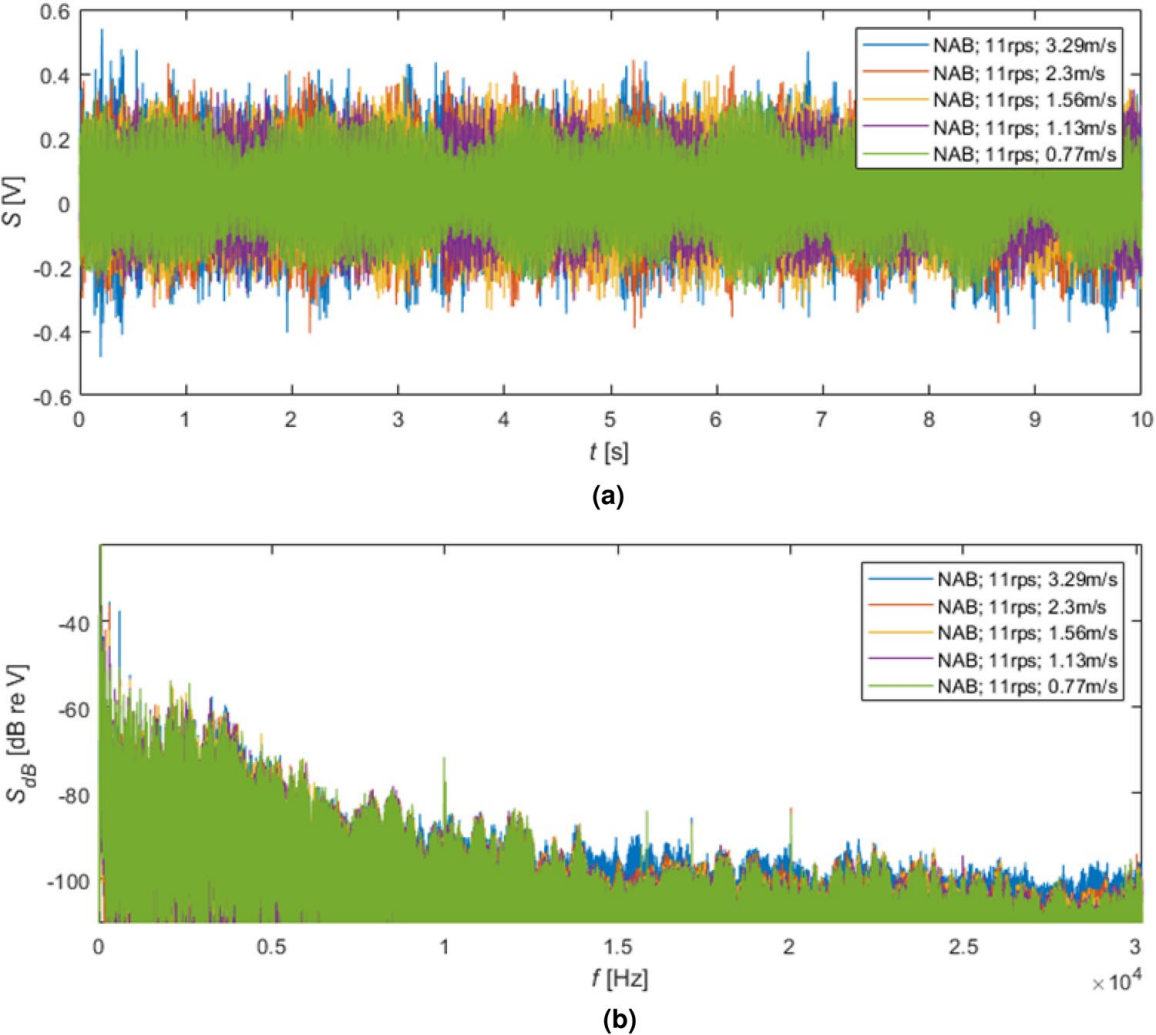
The results of the time and frequency analyses for NAB propeller rotating with speed 11 r/s: (**a**) time signals, (**b**) spectra in dB.

In Figure 6, the time and frequency analysis results for the NAB propeller rotating at 11 r/s for five different water flow speeds were illustrated. In practice, the water flow speed was changed more often, i.e. every smaller range of values, but only five cases were visualized. For the highest speed, i.e. 3.29 m/s, the balance between the produced thrust and damping force of the ambient water was obtained, and that water flow speed was used in further research. This procedure was applied to two propellers and all selected rotating speeds.

As can be seen in Fig. 6a), specific oscillation of the signal envelope was observed. Based on Fig. 6b, the dominant 50 Hz component, likely coming from the power network, was obtained, which causes problems with the evaluation of the spectra for very low frequencies, significantly below 50-100 Hz.

The differences between the noise levels generated during minimal and maximal water flow speeds for the increasing rotational speeds (Fig. 6) can be noticed. Such a phenomenon is visible, especially for higher frequencies, i.e. higher than 1 kHz. Moreover, the increased level of produced noise for lower band, i.e. frequencies lower than 1 kHz, can be seen for the increased rotational speeds of the NAB propeller and corresponding water flow speeds.

**Figure 7:**
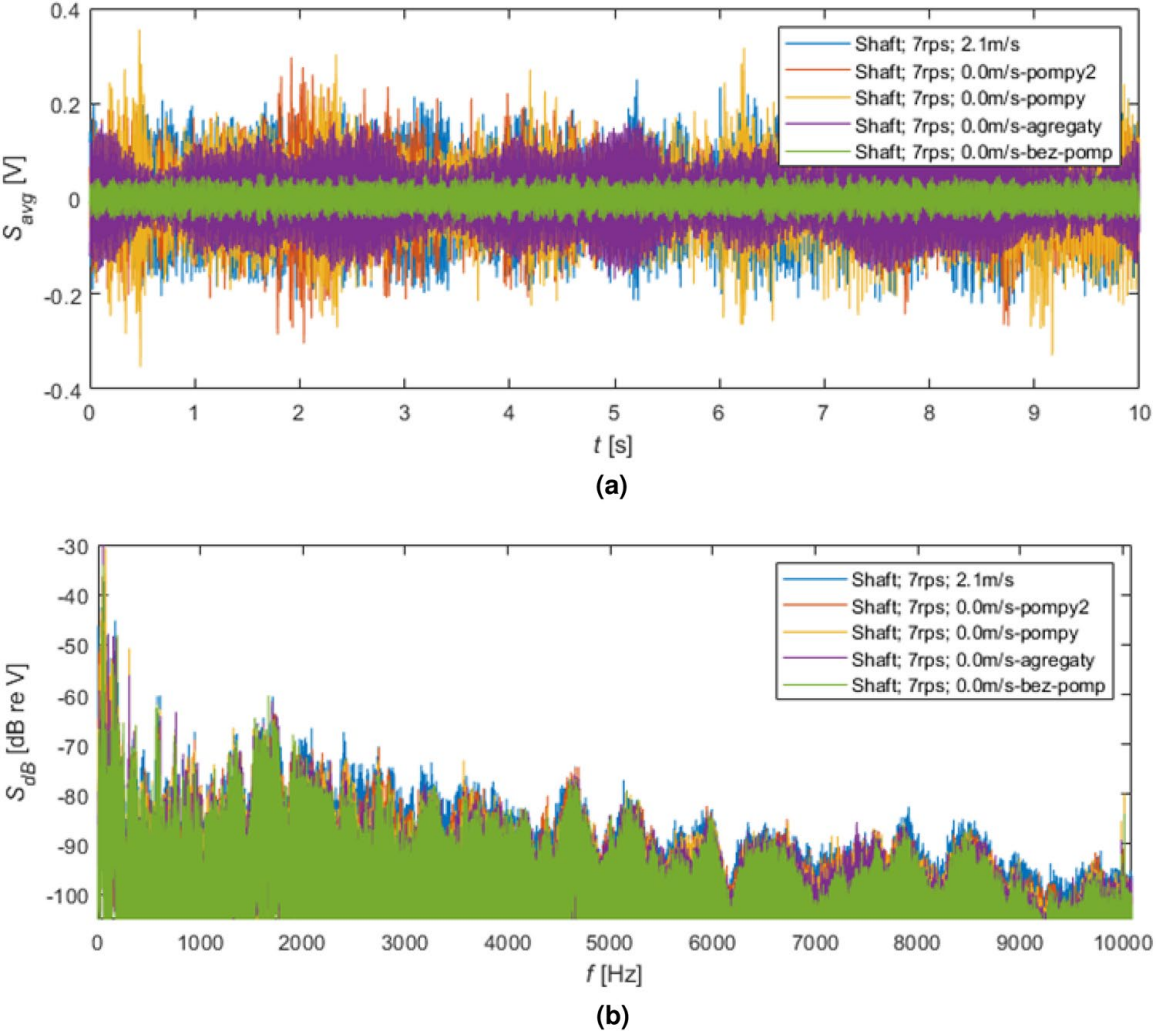
The results of the time and frequency analyses for the shaft rotating without a propeller with speed 7 r/s for the switched on the following tunnel equipment: (**a**) time signals, (**b**) spectra in dB.

In Fig. 7, the effects of switching on the following tunnel subsystems were shown. The lower signal (green lines) was received for the rotating shaft without water flow and no pump operation. The increasing level of noise was obtained by turning on the following subsystems responsible for water flow in the cavitation tunnel, i.e. visualized by violet, orange, and red lines. During the starting of these subsystems, a slowly growing flow of water was observed, i.e. achieving a steady set flow speed demanded several minutes of speeding up the water.

### Seizing noise produced by the shaft without propeller

**Figure 8:**
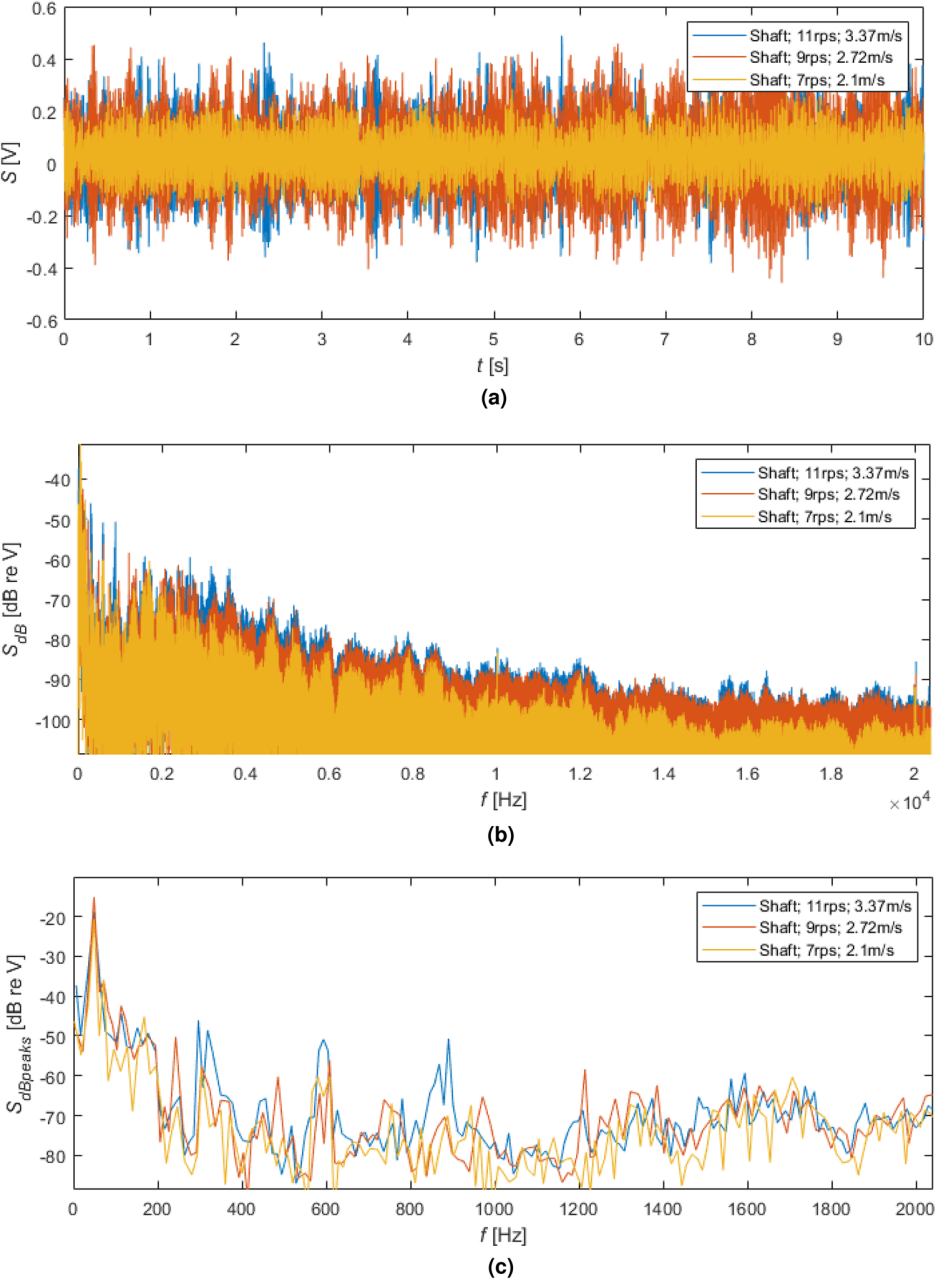
The results of the time and frequency analyses for shaft rotating without propeller with different speeds: (**a**) time signals, (**b**) spectra and (**c**) spectra of signals peaks in dB.

In Fig. 8, the results of the time and frequency analyses for shaft rotating without a propeller with different speeds were illustrated. All the measurements were done for the water flow speeds, guaranteeing balance between produced thrust and hydrodynamic damping force. For the test with the shaft, the speeds obtained for the NAB propeller were accepted. In that case, it is essential to underline that the shaft without a propeller did not generate significant thrust and hydrodynamic damping force. However, the tests with the rotating shaft were important regarding background noise determination and reference of the two examined propellers to zero conditions.

As shown in Fig. 8, the increase in rotational speed gave a visible difference in the signal levels in both time and frequency domains. However, it seems that the difference in the signal levels is larger between 7 r/s and 9 r/s rotational speeds than 9 r/s and 11 r/s ones.

It is worth remembering that the 50 Hz component was also noticed despite the measurement devices (PC, ADC, and receiving hydrophone) being powered from independent and separated electric energy sources. A galvanic connection between the hydrophone case and the stand was possibly the source of that disturbance. Unfortunately, due to this phenomenon, it is hard to evaluate signals in the low-level band.

**Figure 9:**
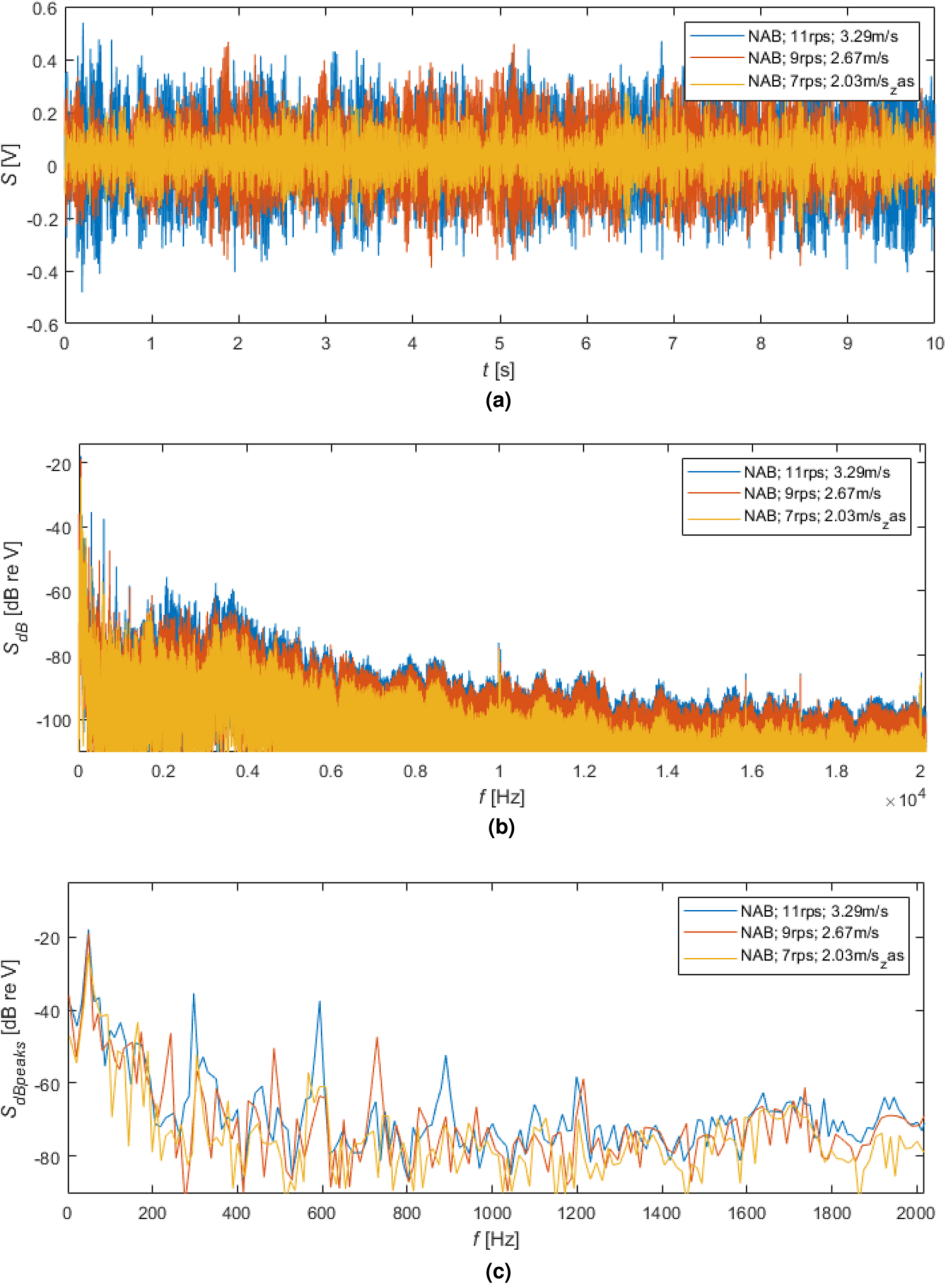
The results of the time and frequency analyses for rotating the NAB propeller with different speeds: (**a**) time signals, (**b**) spectra and (**c**) spectra of signal peaks in dB.

**Figure 10:**
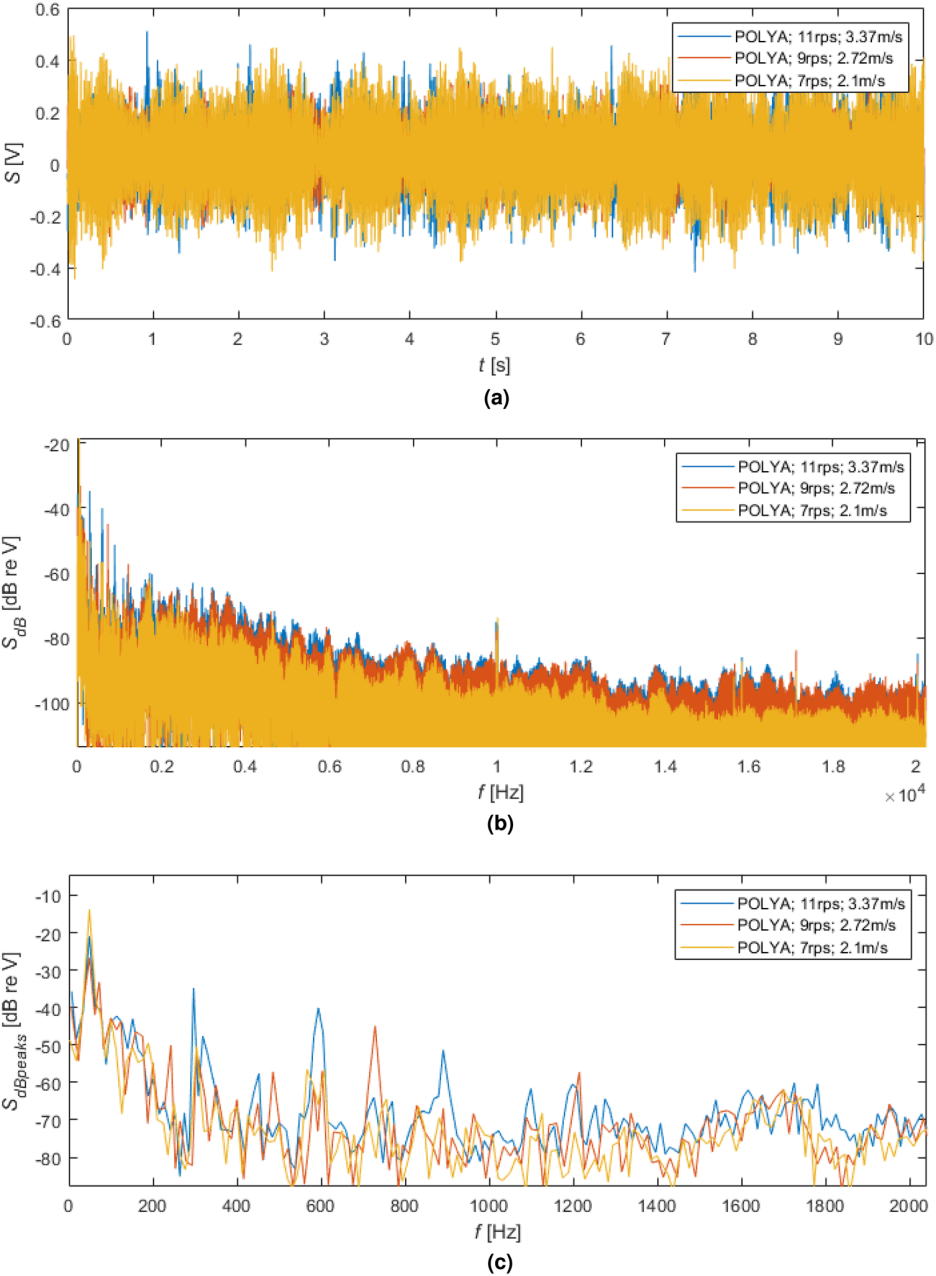
The results of the time and frequency analyses for rotating the NAB propeller with different speeds: (**a**) time signals, (**b**) spectra and (**c**) spectra of signals peaks in dB.

The spectrum visualization in the form of the signal peaks (Fig. 8c) allowed the isolation of important harmonic components. For the largest rotational speed, i.e. 11 r/s, except the mentioned 50 Hz component, harmonics at the following frequencies deserve special attention: approx. 150, 300, 600 and 900 Hz. Such dominant frequencies can be also found in the spectra of the signal peaks for two propellers (Figs. 9c and 10c). For the smaller rotational, i.e. 9 r/s, the dominant harmonics have their amplitudes and frequencies proportionally reduced (Figs. 8c, 9c and 10c). Therefore, they are directly connected by the rotating shaft and/or gearbox.

### Measurement of the NAB propeller’s hydroacoustic signature

In Fig. 9, the results of the time and frequency analyses for rotating the NAB propeller with different rotational speeds were presented in the form of time signals, spectra and additional spectra of signal peaks in dB. Signal peaks are understood as local maxima determined using the findpeaks Matlab function with the MinPeakDistance option equal to 10. The option improves the estimation of the cycle duration by ignoring peaks that are very close to each other. The spectra processed using the findpeaks function enabled us to determine the specific frequencies characterizing the particular spectra.

As shown in Fig. 9, the increase in rotational speed of the NAB propeller gave a visible difference in the signal levels in both time and frequency domains. Similar to the previous case for the shaft without any propeller, the difference in the signal levels is larger between 7 r/s and 9 r/s rotational speeds than between 9 r/s and 11 r/s speeds.

### Seizing hydroacoustic signature of polyamide propeller

In Fig. 10, the results of the time and frequency analyses for rotating the polyamide propeller with different speeds were illustrated. Compared to the previously obtained time signals, signals received for the polyamide propeller for different rotational speeds (Fig.10a) have almost the same amplitude. Based on their spectra (Fig. 10b), different noise levels, especially for higher than 2 kHz frequencies, were received, i.e. increasing with the increasing rotational speeds.

### Comparison

**Figure 11:**
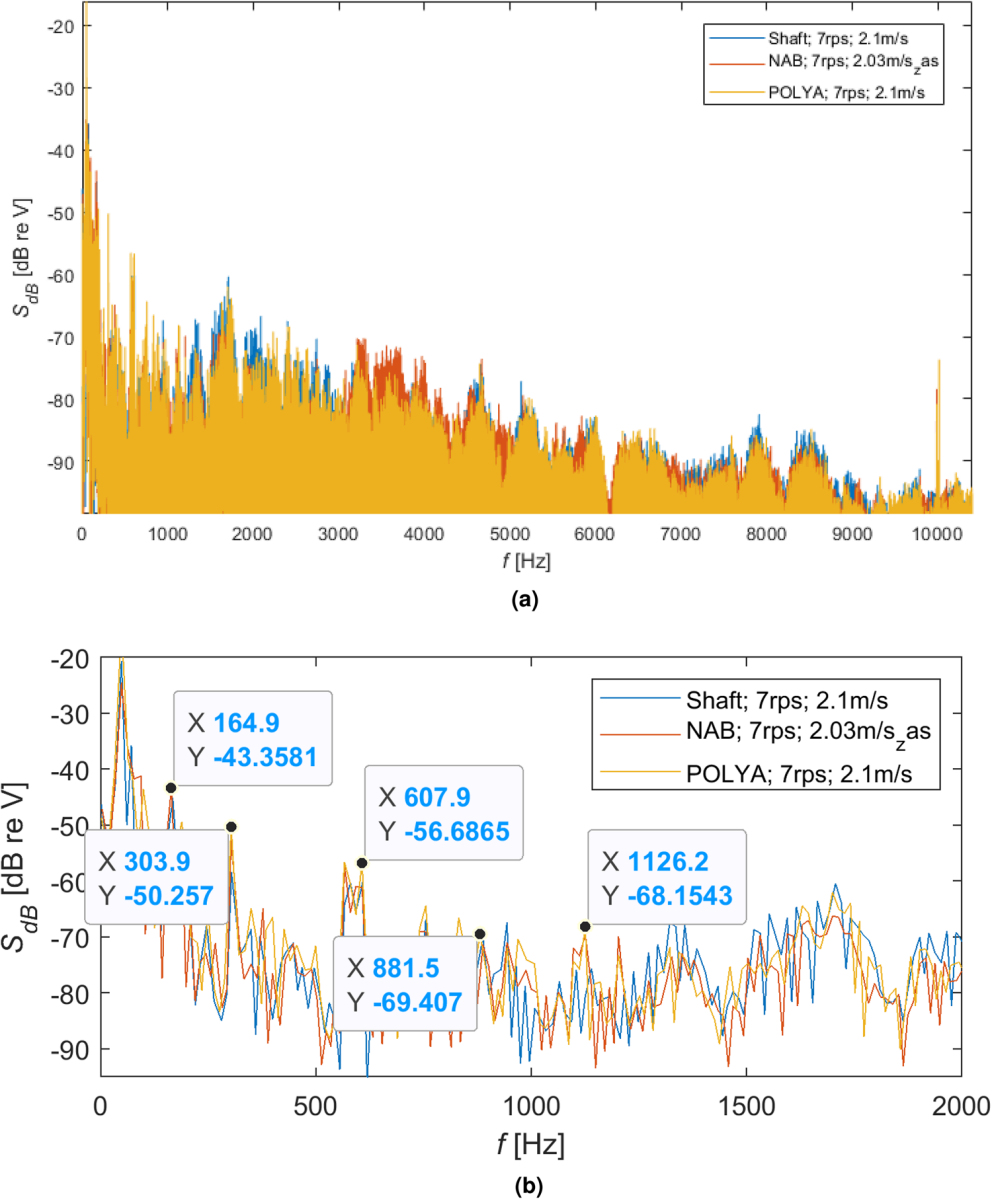
Comparison of different shaft loading rotating with the same speed 7 r/s: (**a**) spectra in dB and (**b**) spectra of signals peaks in dB.

**Figure 12:**
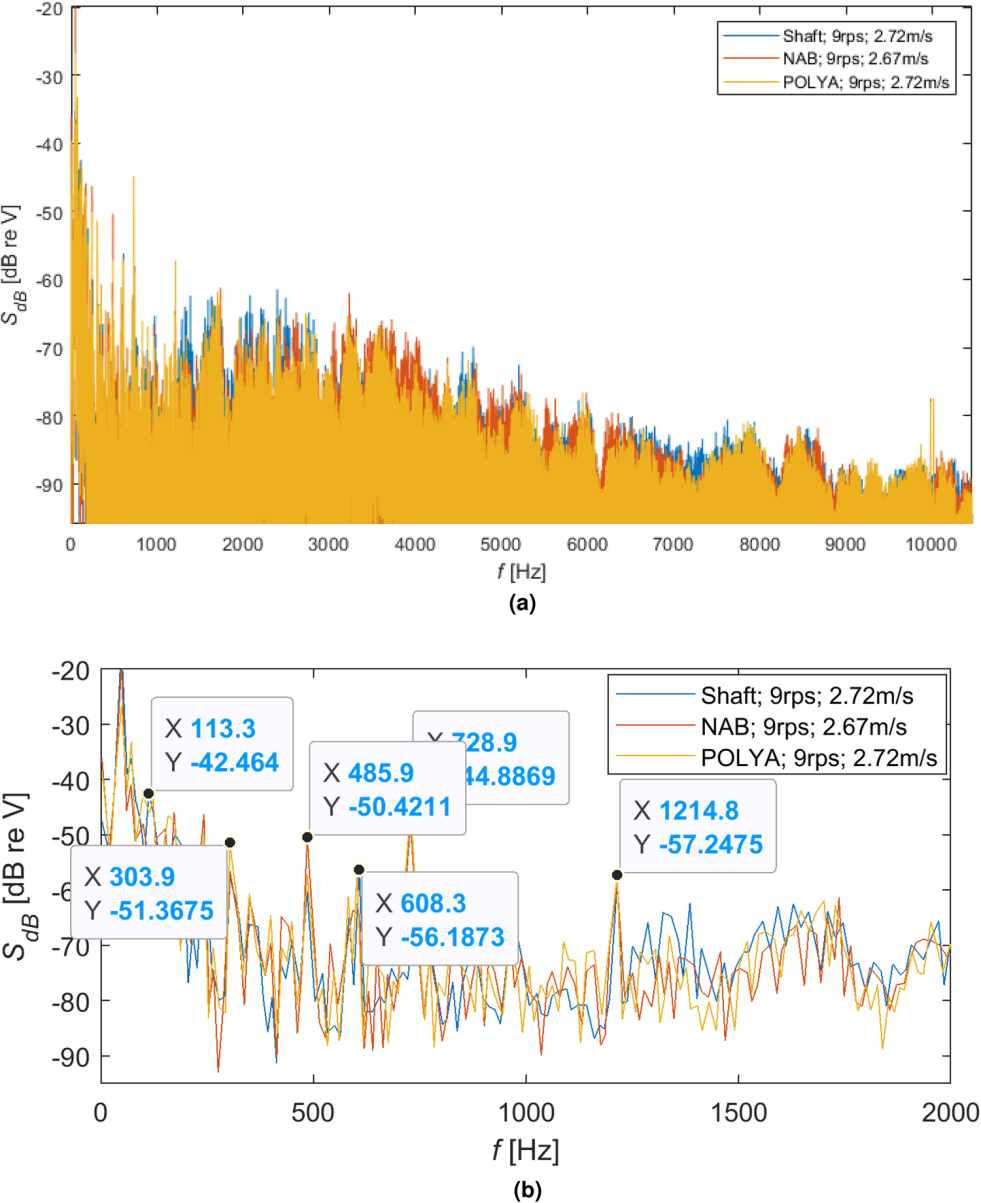
Comparison of different shaft loading rotating with the same speed 9 r/s: (**a**) spectra in dB and (**b**) spectra of signals peaks in dB.

**Figure 13:**
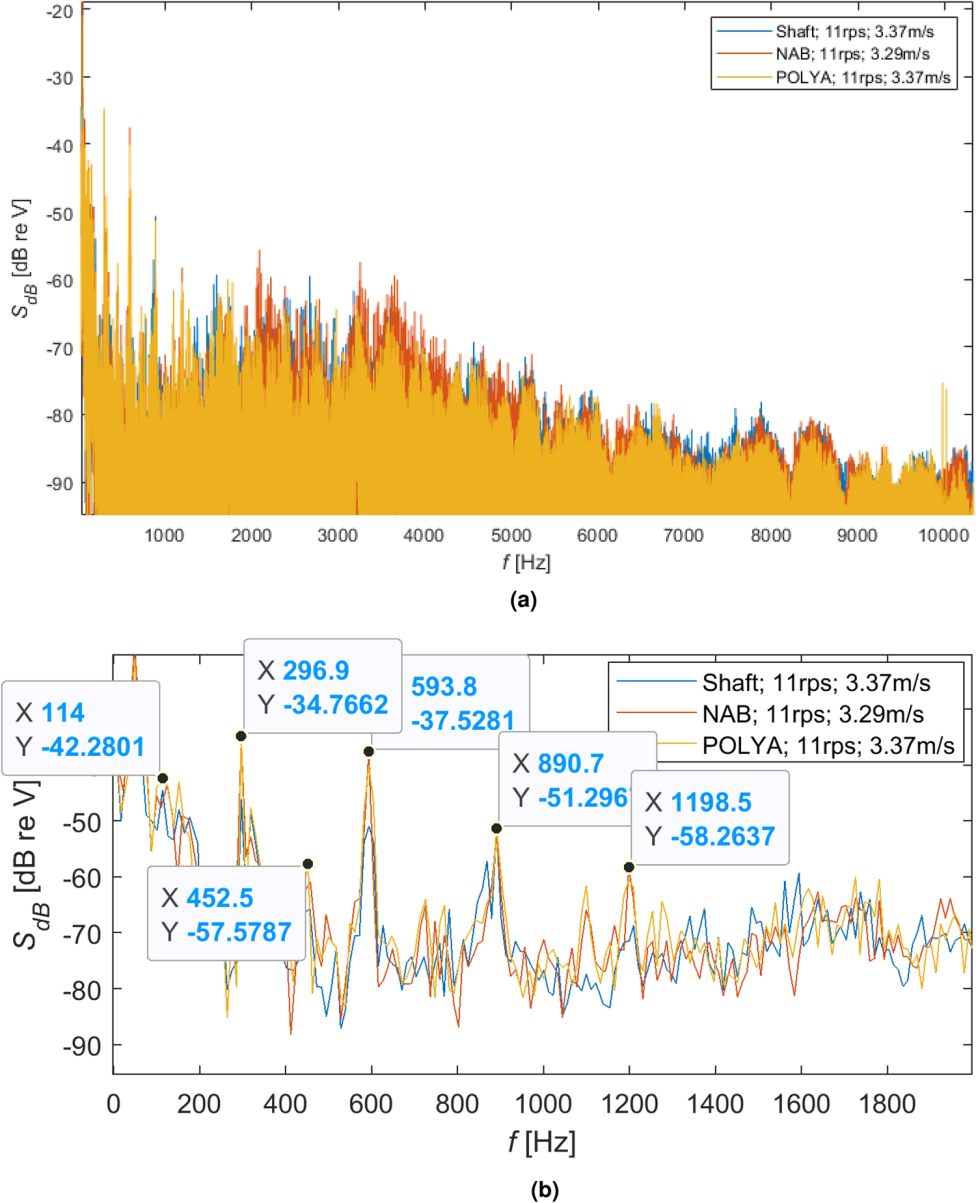
Comparison of different shaft loading rotating with the same speed 11 r/s: (**a**) spectra in dB and (**b**) spectra of signals peaks in dB.

In the following Figs. 11, 12, 13, comparisons of different shaft loading rotating with the same speeds, respectively 7 r/s, 9 r/s and 11 r/s, were presented in the form of spectra and spectra of signals peaks, expressed in dB.

As can be seen for different rotational speeds (Figs. 11b, 12b and 13b), even though the dominant harmonics are not often located at the same frequencies, their amplitudes increase with increasing rotational speed. It should be emphasized that although the harmonics are located at slightly different frequencies, their spread is not too large, i.e. it is acceptable.

Moreover, larger amplitudes can be observed for the NAB propeller than for the flexible propeller and the shaft without a propeller. This effect is less visible for lower rotational speeds of the propeller, although it is clear visible for higher speeds, i.e. 7 r/s and 11 r/s.

Analyzing hydroacoustic signatures visualized in Figs. 11a, 12a, 13a, the following partial conclusions can be formulated:

1. Shaft with an unmounted propeller compared to the polyamide propeller generates higher amplitude for the frequency bands approx. from 1.2 to 3 kHz and from 7 to 9 kHz with almost the same intensity for the increased rotational speeds.
2. Compared to the polyamide propeller, the NAB propeller produces higher amplitude for the frequency bands approx. from 3 to 7 kHz with increasing intensity and also a frequency band for the increased rotational speeds,
3. The polyamide seems to have the lowest amplitude in almost all analyzed frequency bands from 0 to 10 kHz.

Moreover, the analysis of the acoustic emission of the propeller shaft without propellers indicates the highest SPL values in the range from approx. 1kHz to 3kHz. It seems that in the analyzed frequency range, the primary source of emitted acoustic energy is the shaft bearing and not the propeller. This conclusion indicates that no conclusions should be drawn about the acoustic emission of the tested propulsors in the analyzed frequency range.

As shown in Figs. 11b, 12b, 13b, almost the same characteristic frequencies were noted for the different shaft loading but with different amplitudes. It indicates that the shaft with subsystems responsible for the water flow and the mentioned earlier power network disturbance are dominating components of the registered hydroacoustic signals.

To make a more quantitative assessment of the obtained results, the average signal levels $S_{avg}$ of the hydroacoustic signal for different frequency bands from $f_1$ to $f_2$ frequencies were calculated.

The results of calculated $S_{avg}$ for different frequency bands are placed in Table 2.

**Table 2:** The average signal level in dB of the shaft without a propeller, NAB and polyamide propellers for different frequency bands.

Shaft load	Rotational speed	The average signal level in [dB] for the following bands			
	[r/s]	0–1 [kHz]	0–2 [kHz]	2–10 [kHz]	0–100 [kHz]
Without propeller	11	− 87.55	− 86.68	− 90.34	− 104.91
	9	− 86.93	− 85.67	− 92.06	− 105.34
	7	− 90.52	− 88.90	− 97.06	− 111.92
NAB propeller	11	− 86.16	− 85.85	− 90.41	− 105.48
	9	− 88.08	− 87.45	− 92.14	− 106.58
	7	− 89.73	− 89.29	− 97.12	− 111.83
Polyamide propeller	11	− 85.17	− 84.36	− 91.38	− 105.49
	9	− 88.14	− 86.98	− 92.20	− 106.16
	7	− 87.65	− 87.73	− 97.81	− 112.29

As can be observed for the following frequency bands:

1. 0–1 kHz: The polyamide propeller is quitter than NAB propeller by 1–2 dB.
2. 0–2 kHz: The NAB propeller is quitter than polyamide propeller by 1–1.5 dB.
3. 2–10 kHz: The polyamide propeller is quitter than NAB propeller by 0.5–1 dB.
4. 0–100 kHz: The polyamide propeller is quitter than NAB propeller by 0–0.5 dB.

Assuming that on average the polyamide propeller is quieter than the NAB propeller by 1 dB, it can be concluded that the flexible propeller was approx. 12% quieter than the classical one. It should be reminded high level of background noise in that place.

It is worth explaining that the contradictory value of the average signal level was obtained for the frequency band 1-2 kHz. For that band, the rigid propeller was quieter than the elastic propeller. It seems that thrust bearing has the dominant influence on that band. The lower load of the elastic propeller caused the lauder work of the thrust bearing and in consequence, the elastic propeller compared to the rigid one. It is confirmed by the work of a shaft without any propeller, which is even louder than the elastic propeller for the analyzed 1–2 kHz frequency band (Table 3 and Figs. 11a, 12a and 13a.

Table 3 shows the speeds of water flow guaranteeing balance between produced thrust and hydrodynamic damping force for NAB and polyamide propellers. As can be seen, higher speeds were obtained for the polyamide propeller than for the NAB propeller. The relative differences for various rotational speeds are not significant but positive for the polyamide propeller for all cases. Averagely, a 2.58% larger efficiency was achieved for the flexible propeller.

The tests on both propellers were carried out at the constant rotational speed of the propeller shaft in the cavitation tunnel. The controlled parameter was the speed of the water flow, which was set by adjusting the water pump’s speed in the cavitation tunnel. The speed of the water stream was fixed when the dynamometer measuring the propeller’s thrust indicated the value of 0 N. The conducted research showed that for the same rotational speeds of the propellers, the determined speed of the water stream was higher for the flexible propeller. This means its greater energy efficiency, which is also associated with a greater thrust force for a given shaft rotation speed and the possibility of generating greater radial forces on the thrust bearing of the propeller shaft.

**Table 3:** Speeds of water flow guaranteeing balance between produced thrust and hydrodynamic damping force for NAB and polyamide propellers.

Shaft load	Rotational speed	Water flow velocity	Relative difference
	[r/s]	[m/s]	[%]
NAB propeller	11	3.29	− 2.43
	9	2.67	− 1.87
	7	2.03	− 3.44
Polyamide peopeller	11	3.37	2.43
	9	2.27	1.87
	7	2.1	3.44

## Conclusions

This work presents the results of the comparative analysis of the hydroacoustic signatures of NAB and polyamide propellers working with the selected rotational speeds and corresponding flowing water speeds. The experiment compared the new flexible isotropic propeller manufactured from polyamide and the classical one made from metal alloy NAB. The comparison was made mainly regarding produced noise.

In general, the polyamide propeller was approx. 12% quieter than the classical one. Moreover, an average 2.58% larger advance speed for the same rotational speeds was obtained for the new propeller.

The obtained results of the research are promising for future studies on using flexible materials for marine propellers. However, it is worth mentioning that the Nextprop project ended on the 6th Technology Readiness Level, i.e. such an approach requires several additional tests, especially on examining flexible propellers in the real environment, e.g., novel propellers of underwater vehicles. Using tests on underwater vehicles enables us to measure generated noise and consumed electric energy during desired motion. Regarding future research, also fatigue and aging tests are needed to evaluate the commercialization capabilities and potential of the new propellers.

It is worth reminding the high level of the background noise measured in the cavitation tunnel in the CTO, which can be a factor to undermine the conclusion obtained for produced noise. Therefore, it is proposed in future research to design and build a new cavitation tunnel destined for a smaller propeller, e.g. used in AUVs. The tunnel should be placed in a horizontal plane to enable us to turn off all the water flow subsystems for the short period of the hydroacoustic measurement. Another factor that should be considered is properly separating the hydrophone measurement chain from the power network.

Possibly, the research on flexible propellers will be continued in the European Defence Agency in the form of the Nextprop II project. In that case, some of the above-mentioned research directions can be carried out.

## Data Availability

The data that support the findings of this study are available from the European Defence Agency but restrictions apply to the availability of these data, which were used under license for the current study, and so are not publicly available. Data are however available from the authors upon reasonable request and with permission of the European Defence Agency.

## Code availability

The scripts in Matlab for data processing signals received from hydrophones are available from the corresponding author on reasonable request.
